# A dual-layer vector map encryption scheme using 4D hyperchaos and SM4

**DOI:** 10.1038/s41598-026-55081-z

**Published:** 2026-06-06

**Authors:** Mingliang Wang, Jing Wu, Yang Song, Hui Zhang, Xing Zhu

**Affiliations:** 1 College of Computers Science and Cyber Security, Chengdu University of Technology, Chengdu, 610059 China; 2 China Anneng Group Third Engineering Bureau Co., Ltd., Chengdu, 611136 China

**Keywords:** Vector map, 4-D hyperchaotic system, SM4, Dynamic selection, Numerically reversible encryption, Engineering, Mathematics and computing, Physics

## Abstract

Vector map data, as a fundamental component of national geographic information, plays a strategic role in areas such as national security, urban planning, and smart city development. However, with the rapid advancement of information technology, vector map data faces increasing security challenges during transmission and storage. On the one hand, traditional coordinate-scrambling methods are vulnerable to reverse analysis since the numerical values remain essentially unchanged. On the other hand, some existing approaches rely on low-dimensional chaotic systems, which are prone to short periods and dynamical degradation, thereby reducing randomness and security. To address these challenges, this paper proposes a dual-layer encryption method for vector maps that integrates a four-dimensional hyperchaotic system with the national cryptographic algorithm SM4. In this method, the four-dimensional hyperchaotic system generates multiple high-randomness sequences, from which sequences are dynamically selected to scramble coordinates and thereby alter the spatial topological relationships. Furthermore, the chaotic sequences are employed to generate dynamic SM4 keys and initialization vector, enabling deep encryption of coordinate values. Experimental results demonstrate that the proposed method not only effectively protects the three types of vector data–points, polylines, and polygons–but also exhibits significant advantages in resisting brute-force attacks, differential attacks, and low-dimensional chaotic degradation. Overall, it substantially enhances the security and applicability of vector map data during transmission and storage.

## Introduction

Vector map data, as a fundamental component of a nation’s geographic information infrastructure, represents an indispensable strategic asset for both economic development and national defense^1^. It is extensively applied in critical domains such as national security, urban planning, intelligent transportation systems, emergency management, and smart city initiatives^2–5^. Moreover, vector map data often contains highly sensitive information, including national resources, environmental conditions, population distribution, economic indicators, and even military defense details, thereby directly affecting national sovereignty and security^6,7^.

The rapid advancement of cloud computing, the Internet of Things (IoT), and 5G technologies has greatly improved the efficiency of data storage and transmission. However, these developments have also substantially increased the risk of map data leakage^8–10^. Although various countries have introduced policies and regulations to strengthen geographic information security, such measures alone are insufficient to ensure comprehensive protection throughout the data lifecycle. This highlights the urgent need for robust technical mechanisms to safeguard vector map data^11,12^.

In recent years, significant progress has been made in vector map encryption, with several studies incorporating chaotic systems to enhance algorithmic randomness and unpredictability^13^. Tan et al.^14^ proposed an encryption method integrating a two-dimensional Henon–Sine map with RSA, enabling numerically reversible encryption and decryption of multiple types of vector map data. However, this method relies on a low-dimensional chaotic system, which is susceptible to periodicity or degradation in numerical implementations, thereby compromising security^15^. Furthermore, RSA, as an asymmetric encryption algorithm, incurs high computational overhead during both encryption and decryption. Wang et al.^12^ developed a scrambling-based encryption strategy employing a four-dimensional hyperchaotic system. Although high-dimensional chaotic systems can generate highly random sequences, this approach relies solely on scrambling operations and cannot effectively encrypt point data within vector maps, limiting its applicability. Yan et al.^16^ introduced an encryption strategy combining a four-dimensional chaotic system with DNA encoding, which can support encryption of multiple vector map data types. Nevertheless, the DNA-based scheme essentially functions as a coding transformation rather than a robust encryption mechanism, with a relatively limited key space. Additionally, it has not undergone comprehensive cryptographic validation, leaving uncertainties regarding its resistance to known-plaintext attacks and rendering it unsuitable for applications requiring high security.

Despite the aforementioned advances, existing vector map encryption schemes still suffer from several fundamental limitations that remain insufficiently addressed. First, many existing schemes rely solely on chaos-based scrambling as the primary protection mechanism. These approaches focus primarily on permuting coordinate sequences or disrupting spatial topology, while leaving the coordinate values themselves numerically unencrypted. However, as scrambling is traditionally a component for confusion in a complete cryptographic framework, using it in isolation makes the data vulnerable to value-based statistical analysis and partial reconstruction attacks, especially for point-type vector data. Second, cryptography-based methods, although capable of protecting numerical values, often overlook the preservation and concealment of spatial topology. Direct encryption of coordinates may retain topological fingerprints, which can be exploited through structural inference or subgraph matching with public map data. Third, many existing schemes rely on low-dimensional chaotic systems or fixed chaotic sequence usage, which are prone to numerical degradation and predictability in practical implementations, thereby weakening long-term security. These limitations indicate that an effective vector map encryption scheme should simultaneously ensure robust numerical encryption, structural obfuscation, resistance to chaotic degradation, and applicability to heterogeneous vector data types.

To address the above limitations, this paper proposes a dual-layer encryption scheme for vector maps that integrates a four-dimensional hyperchaotic system with a cryptographically strong symmetric encryption algorithm, namely SM4. As China’s national standard for symmetric encryption, SM4 adopts a 128-bit block size and key length and has been extensively deployed in security-critical information systems. Existing cryptanalytic studies demonstrate that SM4 exhibits strong resistance against classical attacks, including differential and linear cryptanalysis^17,18^, making it a reliable choice for protecting sensitive coordinate values.

The scheme incorporates a dynamic chaotic sequence mapping mechanism, which performs coordinate sequence scrambling followed by SM4-based value encryption. Dynamic encryption keys and initialization vectors are generated from chaotic sequences to enable in-depth protection of the coordinate values themselves. This design establishes a dual security framework of “structural scrambling + data encryption,” providing significant advantages in key space, key sensitivity, resistance to statistical analysis, and protection of geometric information.

Moreover, the proposed scheme achieves numerically reversible encryption, ensuring that after decryption, the coordinate values are exactly restored to their original floating-point representations without any precision loss. This property is particularly important for vector map data, as it preserves geometric accuracy and guarantees the practical usability of the decrypted maps in real GIS applications.

The contributions of this paper can be summarized as follows:

1. We identify and address the long-standing limitation of chaotic scrambling-based vector map encryption schemes, which fail to protect coordinate values and cannot effectively encrypt point-type data. By integrating SM4 encryption at the coordinate value level, the proposed method achieves complete numerical protection while maintaining strict reversibility.
2. To overcome the predictability and degradation issues associated with fixed or low-dimensional chaotic systems, a four-dimensional hyperchaotic system with dynamic chaotic sequence selection is introduced. This mechanism adaptively selects scrambling sequences based on coordinate distribution characteristics, significantly enhancing randomness and resistance to statistical and differential attacks.
3. A dual-layer encryption framework is constructed, which simultaneously disrupts spatial topology and encrypts coordinate values. Unlike existing methods that focus on either structure scrambling or numerical encryption alone, the proposed scheme effectively eliminates topological fingerprints while preserving geometric accuracy, thereby achieving a higher level of practical security for heterogeneous vector map data.

## Related work

### Vector map data

**Fig. 1:**
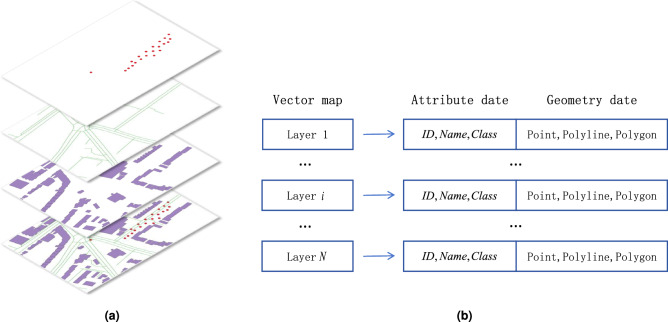
Structure of the vector map: (**a**) Model of the vector map and (**b**) Data components of the vector map.

Vector map data typically comprises multiple data layers, each containing a specific category of geometric objects along with their associated attribute information^19^. Geometric information represents the shape and spatial location of features within a geographic space, commonly including points, polylines, and polygons. Attribute information describes the non-spatial characteristics of geometric objects, such as identification numbers, names, and types^20^. Points denote single-location objects and are zero-dimensional entities, exemplified by points of interest or survey markers. Polylines are formed by sequentially connecting a series of points, constituting one-dimensional geometric objects frequently used to represent linear features such as roads and rivers. Polygons are two-dimensional composite objects created by connecting a series of points to form closed areas, representing planar entities such as buildings, administrative districts, and lakes. As illustrated in Fig. 1a, a vector map model can be decomposed into three data layers: points, polylines, and polygons. Figure 1bdepicts the overall composition of vector map data.

These spatial geometric objects constitute not only the fundamental building blocks of vector maps but also the core elements that must be preserved and processed during data encryption. Specifically, geometric objects such as polylines and polygons inherently possess topological relationships–for example, the sequential ordering of point connections or the boundary and containment relationships between areas. Such topological relationships are considered fundamental spatial characteristics within vector maps. Tøssebro et al.^21^ emphasize that these relationships underpin geospatial queries and analyses and are closely related to data quality and boundary consistency. Consequently, preserving both geometric structures and topological relationships during vector map protection is of paramount importance. Methods for protecting vector maps can be broadly categorized into three types: encryption methods based on traditional cryptography, encryption methods based on chaotic systems, and encryption methods based on mathematical transformations.

### Methods based on traditional cryptography

Traditional cryptography-based approaches for vector map encryption generally treat map data as ordinary binary files and apply standard symmetric or asymmetric encryption algorithms for holistic encryption^15^. Common symmetric algorithms include DES^22^, AES^23^, and SM4^24^, while widely used asymmetric algorithms include RSA^25^, ECC^26^, and SM2^27^. Zhang Shanshan^28^ combined symmetric and asymmetric encryption to secure the entire map file; however, this approach disrupts the original topological structure. In addition, some studies have explored encrypting vector maps at a finer granularity using traditional cryptographic techniques^24,29,30^. For instance, Cong et al.^24^ employed the SM4 algorithm to encrypt coordinate sequences and generated keys via timestamps. During preprocessing, additive offsets were introduced to coordinate values based on feature IDs. These offsets constitute linear transformations, which provide limited obfuscation capabilities and may be vulnerable to known-plaintext attacks, indicating that the overall security of the system still has room for improvement.

### Methods based on chaotic systems

Chaotic systems have been introduced into vector map encryption research due to their high sensitivity to initial conditions and pseudo-randomness, which can enhance system security^31^. Lian-quan^32^ proposed an encryption method based on chaotic dynamics that takes into account both the geometric and topological characteristics of map data. Li et al.^33^ utilized the Henon map to generate chaotic sequences and encrypted data by disrupting the spatial order and neighborhood relationships of coordinate points within the map. These approaches typically rely on low-dimensional chaotic systems to generate keystreams; however, low-dimensional systems are prone to periodic degradation in numerical implementations due to computational precision limitations, which reduces encryption security. To improve the complexity and unpredictability of chaotic sequences, Wang et al.^12^ introduced a hyperchaotic system for scrambling coordinate sequences in vector maps. Although this method enhances the system’s chaotic properties, it does not encrypt the coordinate values themselves, leaving certain security vulnerabilities. Moreover, it cannot encrypt point data within vector maps, limiting the applicability of the algorithm.

### Methods based on mathematical transformations

Mathematical transformation-based methods selectively manipulate geometric objects in vector maps and perform encryption within specific transform domains, constituting a typical selective encryption approach^2,34^. Giao et al.^35^ proposed a geographic information system(GIS) vector map selective encryption method using hybrid transformations. In this approach, vector object sets are first filtered and modified via geometric transformations, and then encryption is performed in the discrete cosine transform (DCT) domain. However, since only key coefficients are encrypted, the geometric contours remain easily exposed, resulting in limited overall security. Bang et al.^36^ simplified polylines features in vector maps and encrypted linepolylines segments using pseudo-random sequences generated by chaotic systems. This method allows flexible control over the amount of encrypted data and improves efficiency. Nevertheless, non-key points are left in plaintext, posing a significant risk of structural information leakage. Wang et al.^37^ mapped vector coordinates to the Haar wavelet domain and introduced Gaussian random perturbations to effectively encrypt coordinate points along with their spatial structures. However, this approach relies on perturbing transform coefficients rather than performing strong encryption, offering limited resistance to attacks and potentially introducing precision errors.

Overall, holistic encryption methods tend to disrupt the topological structure of vector maps, while scrambling approaches only disturb the order of points or adjacency relationships, leaving the coordinate values unprotected and incapable of processing point data. Fine-grained encryption can conceal coordinate values, but it often overlooks topological relationships. The retained topological information itself contains significant clues about the shapes of geographic features. Attackers can exploit these topological characteristics or perform subgraph matching with publicly available map data to infer feature types or achieve a certain degree of localization. Therefore, numerical encryption alone cannot fully prevent structure-based reconstruction or identification attacks.

To address this, this paper proposes a dual-layer encryption scheme for vector maps that integrates a four-dimensional hyperchaotic system with the SM4 algorithm. In this scheme, coordinate sequences are first scrambled at the structural level to sever topological fingerprints at their source. Subsequently, coordinate values are encrypted using SM4, achieving a dual effect of structural disruption and numerical encryption, thereby enhancing randomness, security, and applicability.

## Methodology

### Four-dimensional hyperchaotic system

**Fig. 2:**
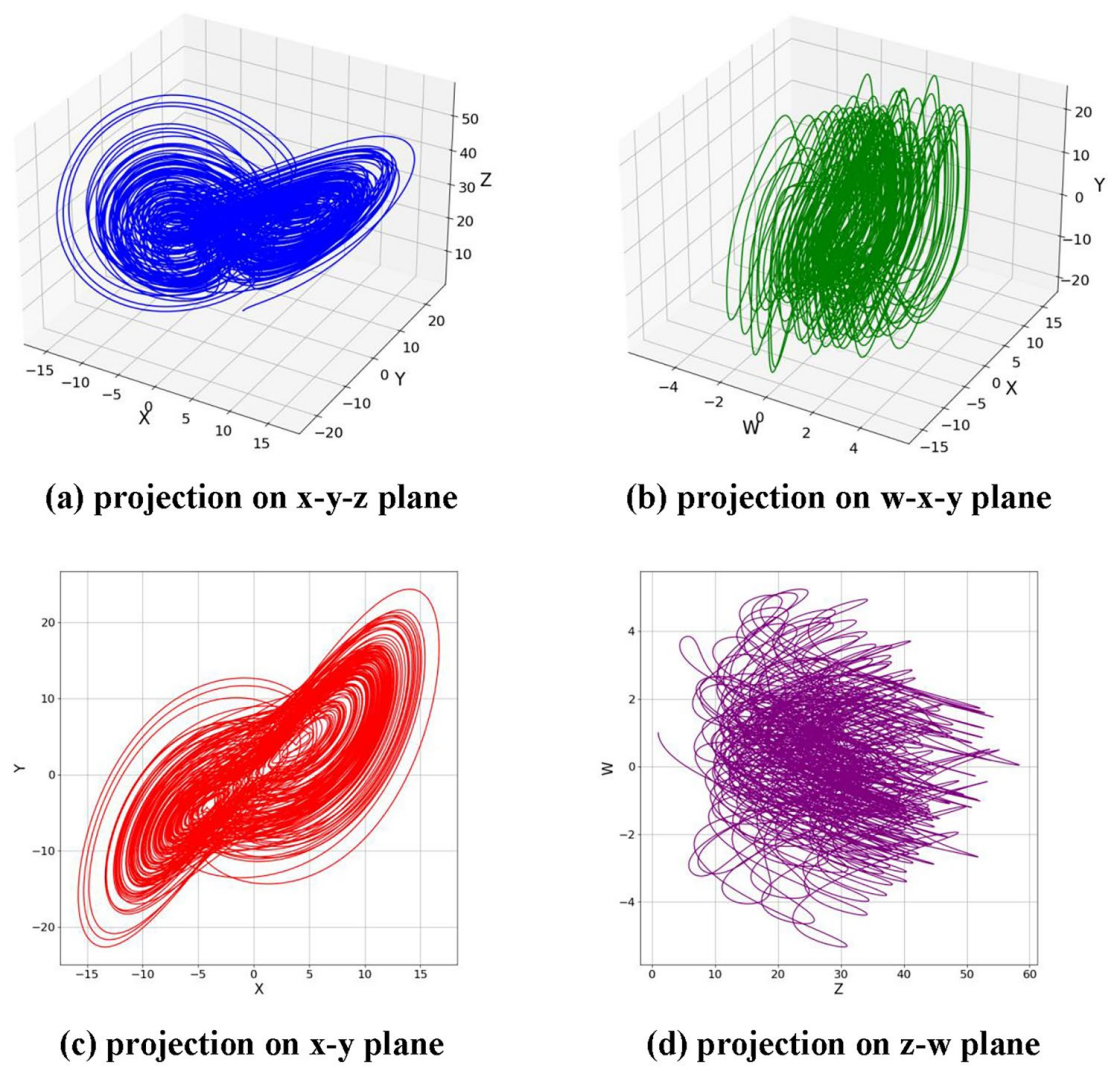
Attractor of the four-dimensional chaotic system.

Chaos refers to a bounded, deterministic, yet unpredictable behavior arising in certain nonlinear dynamical systems. This behavior is fundamentally characterized by its sensitive dependence on initial conditions, topological transitivity, and long-term aperiodicity^38^. However, when implemented computationally, simple discrete chaotic maps often suffer from rapid dynamical degradation due to finite floating-point precision, leading to short-period sequences^15^. Furthermore, while native high-dimensional discrete maps exist, their relatively simple algebraic structures can leave them vulnerable to parameter estimation and algebraic attacks^39^. To mitigate these vulnerabilities, this study employs a four-dimensional continuous hyperchaotic system^40^. Although integrating this system via the second-order Runge-Kutta (RK2) method effectively discretizes it into a computational model, this multi-stage numerical approximation introduces deep algebraic nesting and complex non-linear variable coupling, since each iteration step contains recursively embedded intermediate computations and simultaneous interactions among multiple state variables. This resulting intricate discrete mapping not only expands the state space to delay precision-induced degradation, i.e., the gradual transition of chaotic trajectories into short-period or repetitive states caused by finite computational precision, but also significantly enhances the cryptographic resistance against phase-space reconstruction and mathematical cryptanalysis. The continuous system is defined in Eq. 1:

1 $$\begin{aligned} {\left\{ \begin{array}{ll} \dot{x} = a(y - x) \\ \dot{y} = bx - y + ew - xz \\ \dot{z} = -cz + xy + x^2 \\ \dot{w} = -dy \end{array}\right. } \end{aligned}$$

where, *x*, *y*, *z*, *w* are the state variables, and *a*, *b*, *c*, *d*, *e* are the system parameters. When *a=10, b=28, c=8/3, d=1, and\ e=16*, the system exhibits hyperchaotic behavior^40^. The attractor of this four-dimensional chaotic system with the initial values (1, 1, 1, 1) is shown in Fig. 2:

### Generation of initial values for the chaotic system

In this subsection, the initial values $u_x, u_y, u_z, u_w$ of the four-dimensional chaotic system are derived using the SM3 cryptographic hash function^41^, which integrates both the vector map information and the user-provided secret key. By exploiting the strong diffusion and sensitivity properties of SM3, the resulting initial values are made highly data-dependent, thereby enhancing the unpredictability and security of the chaotic system. The detailed procedure is described as follows:

*Step 1:* The file size (in KB) and the total number of feature points of the original vector map are individually input into the *SM*3 algorithm. An XOR operation is then applied to obtain a 256-bit hash value *H*, which is divided into 32 groups of 8 bits each, denoted as $h_1,h_2,...,h_{31},h_{32}$. The rationale behind this design is to uniformly map the high-entropy hash results into multiple initial parameters of the 4D hyperchaotic system. This establishes a strong correlation between the data characteristics and the initial values, ensuring that the encryption process is highly sensitive to the plaintext, which effectively resists potential differential attacks.:

2 $$\begin{aligned} H = \textrm{SM3}(\textrm{FileSize}) \oplus \textrm{SM3}(|V|) \end{aligned}$$

3 $$\begin{aligned} h_1 \parallel h_2 \parallel \cdots \parallel h_{31} \parallel h_{32} = H \end{aligned}$$

where, |*V*| represents the total number of vertices in the vector map, and $\text {SM3}()$ denotes the $\textrm{SM3}$ hash function.

*Step 2:* The user key is defined as a 128-bit value. To further enhance resistance against repeated-key attacks, a 128-bit random nonce *R* is introduced into the initialization process. Combining this key with the $h_i$ values computed in Step 1, a chained iterative process of 32 rounds is performed to produce a value $U_{32}$. This iterative structure employs a forward feedback mechanism, where the output of each round serves as the input for the subsequent round, thereby achieving multi-layered information mixing. The specific process is as follows:

4 $$\begin{aligned} {\left\{ \begin{array}{ll} U_1 = \textrm{SM3}(h_{32} \parallel K \parallel H \parallel R) \\ U_2 = \textrm{SM3}(h_{31} \parallel K \parallel U_1) \\ \;\;\;\;\;\;\;\vdots \\ U_{32} = \textrm{SM3}(h_1 \parallel K \parallel U_{31})\\ \end{array}\right. } \end{aligned}$$

After 32 rounds of iterative operations, the final output value $U_{32}$ is obtained, which is then divided into eight 32-bit groups, denoted as $e_1,e_2,...,e_8$:

5 $$\begin{aligned} e_1 \parallel e_2 \parallel \cdots \parallel e_8 = U_{32} \end{aligned}$$

where, *K* represents the user key, || represents the byte concatenation operator.

*Step 3:* A factor *a* is computed as shown in Eq. 6. This adjustment factor is introduced to expand the parameter variation range and enhance the system’s sensitivity to datasets of different scales. To ensure that the initial values meet the chaotic system’s requirement for high sensitivity to initial conditions, XOR operations, modular operations, and floor operations are applied to the $e_1,e_2,...,e_8$ values from Step 2, together with selected feature data from the vector map. On this basis, the initial values $u_x, u_y, u_z, u_w$ for the 4D hyperchaotic system are constructed via normalization, as defined in Eq. 7. This construction mechanism creates a rigorous, intrinsic link between the chaotic trajectories and the vector map data. Specifically, the avalanche effect of the SM3 hash (from Step 2) ensures that any infinitesimal perturbation in the map data results in a massive change in the initial values, which are then exponentially amplified by the system’s positive Lyapunov exponents, leading to entirely divergent chaotic orbits.

6 $$\begin{aligned} a = \text {mod}(|V|, 100) + 1 \end{aligned}$$

7 $$\begin{aligned} {\left\{ \begin{array}{ll} \begin{aligned} u_x = \frac{a}{|V| \times |F|} \Big ( (e_1 \oplus e_3 \oplus e_5 \oplus e_7) + \text {mod}(e_2 \times e_4, |H|) \Big ) - \left\lfloor \frac{a}{|V| \times |F|} \Big ( (e_1 \oplus e_3 \oplus e_5 \oplus e_7) + \text {mod}(e_2 \times e_4, |H|) \Big ) \right\rfloor \end{aligned} \\ \begin{aligned} u_y = \frac{a}{|V| \times |F|} \Big ( (e_2 \oplus e_4 \oplus e_6 \oplus e_8) + \text {mod}(e_3 \times e_5, |H|) \Big ) - \left\lfloor \frac{a}{|V| \times |F|} \Big ( (e_2 \oplus e_4 \oplus e_6 \oplus e_8) + \text {mod}(e_3 \times e_5, |H|) \Big ) \right\rfloor \end{aligned} \\ \begin{aligned} u_z & = \frac{a}{|V| \times |F|} \Big ( (e_3 \oplus e_5 \oplus e_7 \oplus e_1) + \text {mod}(e_4 \times e_6, |H|) \Big ) - \left\lfloor \frac{a}{|V| \times |F|} \Big ( (e_3 \oplus e_5 \oplus e_7 \oplus e_1) + \text {mod}(e_4 \times e_6, |H|) \Big ) \right\rfloor \end{aligned} \\ \begin{aligned} u_w & = \frac{a}{|V| \times |F|} \Big ( (e_4 \oplus e_6 \oplus e_8 \oplus e_2) + \text {mod}(e_1 \times e_7, |H|) \Big ) - \left\lfloor \frac{a}{|V| \times |F|} \Big ( (e_4 \oplus e_6 \oplus e_8 \oplus e_2) + \text {mod}(e_1 \times e_7, |H|) \Big ) \right\rfloor \end{aligned} \end{array}\right. } \end{aligned}$$

where |*V*| denotes the total number of vertices, |*F*| denotes the total number of features, |*H*| is the length of the hash value *H*, *⊕* represents the XOR operation, $\text {mod}(a,b)$ denotes the modulo operation of a modulo b, and $\lfloor \cdot \rfloor$ indicates the floor operation. The resulting $u_x,u_y,u_z,u_w$ serve as the initial values for the four-dimensional chaotic system.

The pseudocode for generating the initial values of the four-dimensional chaotic system is shown in Algorithm 1.

**Algorithm 1:**
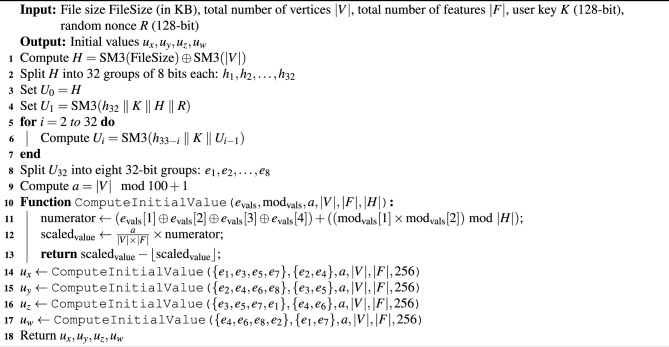
Generation of initial values for the chaotic system.

### The proposed dual-layer encryption scheme

**Fig. 3:**
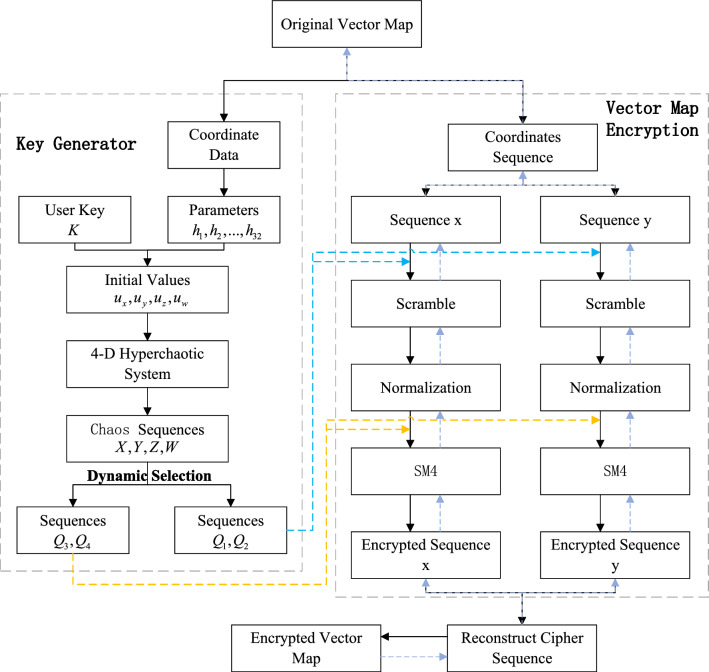
The flowchart of proposed method.

This solution adopts a dual-layer architecture of “structural scrambling + numerical encryption”. The overall process is illustrated in Fig. 3.

*Step 1: extraction of coordinate sequences*

The *x* and *y* components of all coordinates are extracted from the original vector map and stored in the arrays $x_{\textrm{coords}}$ and $y_{\textrm{coords}}$, respectively. During this extraction process, the start and end indices of each feature object within the coordinate sequence are also recorded. This indexing structure enables the precise reconstruction of the original geometric shapes of the vector map after coordinate transformations, such as encryption and decryption.

*Step 2: Generation of chaotic sequences*

Prior to scrambling the coordinate sequences, input the initial values $u_x,u_y,u_z,u_w$ into Eq. 1 to compute the chaotic sequences. Considering the stringent requirements for sequence precision and the constraints of numerical computational efficiency, the second-order Runge-Kutta (RK2) method is employed to solve the differential equations with an integration step size of 0.001. This approach effectively balances numerical stability with computational complexity. To ensure the system fully enters a hyperchaotic state and to eliminate the influence of initial transients on the sequence randomness, the system is iterated *n* times, where *n* is computed according to Eq. 8.

8 $$\begin{aligned} n = 1000 \times \lfloor \mathrm {log_2}(|V| +1 ) \rfloor +|V| \end{aligned}$$

where $\lfloor \cdot \rfloor$ denotes the floor operation. After completing the iterations, only the final segment of length |*V*| is retained as the effective output. This discarding process ensures that the resulting sequences exhibit mature hyperchaotic characteristics. Consequently, four sequences *X*, *Y*, *Z*, *W* of length |*V*| are obtained, providing a high-quality candidate pool for the subsequent dynamic scrambling operations.

*Step 3: Dynamic selection of chaotic sequences for scrambling*

To enhance the unpredictability and data-dependency of the algorithm, a dynamic selection mechanism for chaotic sequences is introduced during the scrambling stage instead of using fixed sequences. First, a chaotic sequence mapping dictionary is constructed, as shown in Eq. 9. Specifically, the sequences used for scrambling are dynamically determined based on the statistical characteristics of the original coordinate data. When scrambling the coordinate sequence *x*, the value $Q_x$ is calculated using Eq. 10, and $Q_x$ is then used as a key to retrieve the corresponding chaotic sequence from the mapping dictionary. This sequence is denoted as $Q_1$. Next, when scrambling the coordinate sequence *y*, the value$Q_y$ is calculated according to Eq. 11. To ensure that the *x* and *y* coordinate sequences do not use the same chaotic sequence, the value of $Q_y$ is updated using Eq. 12. The updated $Q_y$ is then mapped to the corresponding chaotic sequence from the dictionary, denoted as $Q_2$. This process ensures that the two coordinate sequences are processed using distinct chaotic sequences. Finally, the remaining two chaotic sequences are denoted as $Q_3$ and $Q_4$, which are reserved for the subsequent derivation of the SM4 key and IV.

9 $$\begin{aligned} \text {index}_{\text {map}} = \{0 : X, 1 : Y, 2 : Z, 3 : W\} \end{aligned}$$

10 $$\begin{aligned} Q_x = \textrm{floor}( \text {mod}(\max (x_{\text {coords}}) - \min (x_{\text {coords}}), 4) ) \end{aligned}$$

11 $$\begin{aligned} Q_y = \textrm{floor}( \text {mod}(\text {mean}(y_{\text {coords}}), 4) ) \end{aligned}$$

12 $$\begin{aligned} Q_y = \text {mod}(Q_y + 1, 4) \end{aligned}$$

where $\mathrm {max(\cdot )}$ represents the maximum operation, $\mathrm {min(\cdot )}$ the minimum operation, $\mathrm {mean(\cdot )}$ the average operation, and $\mathrm {floor(\cdot )}$ the floor function.

*Step 4: Index calculation of chaotic sequences and scrambling of coordinate sequences*

Since the chaotic sequences generated by the hyperchaotic system consist of double-precision floating-point numbers, their dynamical variations within a specific range are more significantly manifested in the least significant bits (LSBs) of the fractional part. To effectively capture these high-sensitivity variations and eliminate the short-term correlations inherent in the higher-order bits, a decimal-point shifting (amplification) strategy is employed. By scaling the chaotic values, the highly random information residing in the deeper decimal places is shifted to the integer part, which is then extracted via modular operations. This process, commonly referred to as least significant bit extraction in chaotic cryptography, significantly enhances the statistical randomness of the resulting index sequences and ensures that the scrambling process is extremely sensitive to the chaotic trajectories. The mapping indices are computed as follows:

13 $$\begin{aligned} {Q_{\textrm{index1}}} = \bmod ({Q_1} \times {10^n},N) \end{aligned}$$

here, $Q_i$ denotes the chaotic sequence used for scrambling, *n* represents the number of decimal places of elements in $Q_1[i]$, *N* is the number of elements in $Q_1$, and $Q_{\text {index1}}$ denotes the generated index sequence.

The detailed scrambling process is defined by the corresponding equations:

14 $$\begin{aligned} {x_{\textrm{coords}}}[i] \leftrightarrow {x_{\textrm{coords}}}[{Q_{\textrm{index1}}}[i]] \end{aligned}$$

Swap the value at position *i* in the coordinate sequence with the value at the corresponding index to complete the scrambling of the the $x_{\textrm{coords}}$. Similarly, the $y_{\text {coords}}$ is scrambled using the same procedure as that of $x_{\text {coords}}$.

The detailed pseudocode of the coordinate sequence scrambling procedure is provided in Algorithm 2.

**Algorithm 2:**
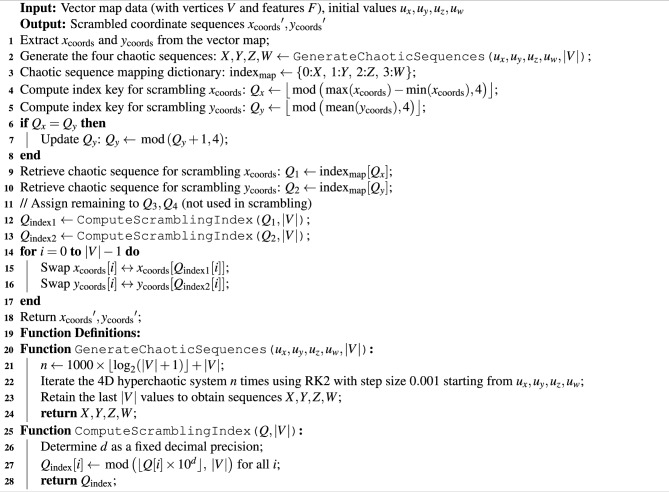
Coordinate sequence scrambling.

**Step 5: SM4 encryption of coordinate sequences**

The scrambling operation alters the ordering of the original coordinate sequence and disrupts its spatial topology. However, scrambling alone only changes the organization of the data without encrypting the coordinate values themselves, which means that the data still faces the risk of being reconstructed through analysis. To address this issue, the SM4 encryption algorithm is introduced to perform point-by-point encryption on the scrambled coordinate sequence. In this process, SM4 ensures the numerical security of the coordinate values, while scrambling guarantees the perturbation of the topological relationships. The combination of these two mechanisms not only enhances the overall encryption strength but also ensures that the ciphertext map exhibits significant differences from the original map in both numerical values and topological structures, while preserving the organizational structure of the vector data. The detailed encryption procedure is described as follows.

The remaining two chaotic sequences, $Q_3$ and $Q_4$, are used to compute the SM4 encryption key and the initialization vector (IV). Both the SM4 key and IV are 16 bytes in length. To enhance the dynamic properties and data-dependency of the cryptographic parameters, the key and IV are derived from the chaotic trajectories through a weighted aggregation mechanism. Each byte calculated as follows:

15 $$\begin{aligned} \textrm{sm4}_{\textrm{key}}[i] = {\mathop {\textrm{floor}}} (\bmod (\sum \limits _{j = i}^{|V|:S} {(({Q_3}[j] \times } {Q_4}[|V| - j - 1]) \times (j + 1) \times 256),256)) \end{aligned}$$

16 $$\begin{aligned} \textrm{iv}[i] = {\mathop {\textrm{floor}}} (\bmod (\sum \limits _{j = i}^{|V|:S} {(({Q_3}[j] \times } {Q_4}[|V| - j - 1]) \times (|V| - j) \times 256),256)) \end{aligned}$$

where |*V*| denotes the length of the chaotic sequence, $i \in \{0,1,\dots ,15\}$ is the byte index of the SM4 key or IV, $j \in \{\, i + kS \mid k \in \mathbb {Z}_{\ge 0},\; i + kS < |V| \,\}$ is the sampling index, $S = \lfloor |V|/16 \rfloor$ is the sampling interval used for uniform aggregation, $\bmod (\cdot )$ denotes the modulo operation, and $\text {floor}(\cdot )$ denotes the floor operation.

Before applying the SM4 encryption, the scrambled coordinate sequences are first normalized. Due to the limited precision of computer arithmetic, precision loss may occur during computation. To ensure numerically reversible encryption and decryption, the coordinate sequences are scaled up and the decimal point positions are recorded. This normalization is performed in decimal representation. Taking $x_{\text {coords}}'$ as an example:

17 $$\begin{aligned} {x_{\textrm{coords}}}''[i] = {x_{\textrm{coords}}}'[i] \times {10^{(n + 2)}} + \mathrm {decimal^{pos}} \end{aligned}$$

where $x_{\text {coords}}'$ denotes the scrambled sequence, $x_{\text {coords}}''$ denotes the normalized sequence, *n* records the number of decimal places, and $\text {decimal}^{pos}$ specifies the decimal point position.

The normalized sequence $x_{\text {coords}}''$ is then encrypted using SM4:

18 $$\begin{aligned} {x_{\textrm{coords}}}_{\_\textrm{encrypted}} = \textrm{SM4}({x_{\textrm{coords}}}'',\textrm{sm4}_{\textrm{key}},IV) \end{aligned}$$

The encryption procedure for the $y_{\text {coords}}$ sequence is identical to that of $x_{\text {coords}}x$. Finally, the encrypted coordinate sequences ${x_{\textrm{coords}}}_{\_\textrm{encrypted}}$ and ${y_{\textrm{coords}}}_{\_\textrm{encrypted}}$ are recombined according to the start and end indices of each object in the vector map, producing the final encrypted vector map.

By integrating a dual-layer mechanism of scrambling and numerical encryption, the proposed algorithm effectively disrupts spatial topological relationships while performing deep encryption on coordinate values. This ensures that the ciphertext map exhibits high confusion characteristics across both structural distribution and numerical layers. Meanwhile, as the original index structure is completely preserved, the decryption stage can precisely restore the forms of the original geometric objects, thereby achieving a seamless integration of robust security and perfect reversibility.

The pseudocode for the SM4 encryption of coordinate sequences is provided in Algorithm 3.

**Algorithm 3:**
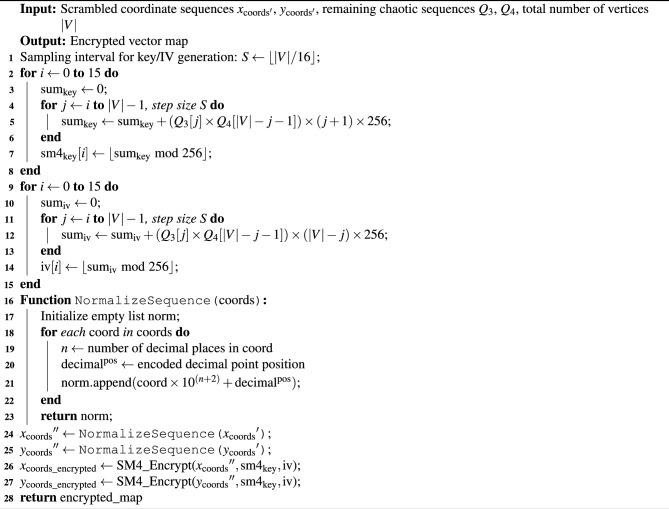
SM4 encryption of coordinate sequences.

### Decryption process

The decryption process is essentially the inverse of the encryption procedure. First, chaotic sequences are regenerated using the chaotic system. These sequences are then employed to guide the SM4 decryption of the ciphertext sequence, producing the scrambled coordinate data. Next, the inverse scrambling operation is applied to restore the original ordering of the coordinates. Finally, the recovered coordinate sequences are recombined according to the recorded indices of each object, yielding the decrypted vector map.

## Experiments

In the experimental section, different types of vector data were used to validate the feasibility of the proposed encryption scheme. The vector datasets employed in this study include three data types: points, polylines, and polygons, which were obtained from OpenStreetMap (https://download.geofabrik.de/). The detailed specifications of the datasets are provided in Table 1.

**Table 1:** Detailed information of vector data.

Dataset	Data layer	Data type	Size (KB)	Vertex count	Feature count
Dataset A	Sichuan nature	Point	184	6,713	6,713
	Sichuan railway	Line	2,882	122,026	17,833
	Sichuan buildings	Polygon	20,925	937,803	114,588
	Sichuan waterways	Line	20,996	1,252,413	26,079
Dataset B	London nature	Point	5,186	189,656	189,656
	London railway	Line	2,566	111,773	14,987
	London buildings	Polygon	174,629	7,512,252	1,046,673

### Encryption and decryption visualization

**Fig. 4:**
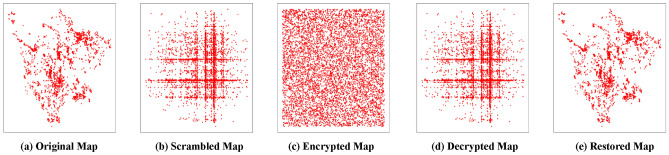
Encryption and decryption results of point data.

**Fig. 5:**
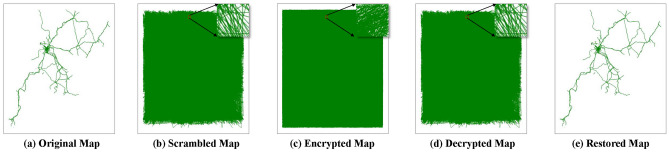
Encryption and decryption results of line data.

**Fig. 6:**
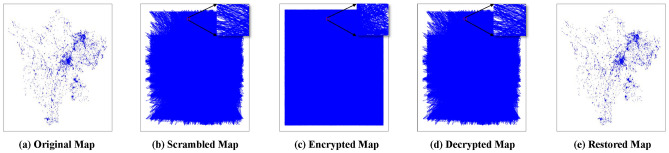
Encryption and decryption results of polygon data.

The system was developed in Python and executed on a Windows 11 environment, equipped with an AMD Ryzen 7 5800H processor and 16GB of RAM. The proposed encryption scheme was applied to vector data to perform both encryption and decryption operations, with the results visualized in Figs. 4, 5, 6. Specifically, Fig. 4a–e illustrate the original map, scrambled map, encrypted map, decrypted map, and restored map for vector point data representing natural features in Sichuan Province. Figure 5a–e show the corresponding results for vector polyline data of railway routes. Figure 6a–e present the results for vector polygon data of buildings.

From the visualizations, it is evident that the encrypted vector maps exhibit strong chaotic characteristics, with coordinates distributed in a highly random manner, effectively preventing the extraction of meaningful information from the original maps. The proposed method demonstrates robust and consistent encryption performance across point, polyline, and polygon spatial data, highlighting its applicability for diverse vector map types.

### Encryption security

#### Chaotic analysis of chaotic sequences

The chaotic properties of a sequence directly affect the security of an encryption algorithm. High randomness and strong chaotic behavior are critical in cryptographic systems. To verify the chaotic performance of the generated sequences, the root mean square error (RMSE) is employed. The initial state of the chaotic system is set as $(u_x, u_y, u_z, u_w)$. A univariate perturbation approach is adopted, where a micro-perturbation of magnitude $10^{-8}$ is applied individually to each of the four initial state variables–$u_x, u_y, u_z$, and $u_w$–while keeping the other three initial values constant. The RMSE values between the corresponding chaotic sequences are calculated using Eq. 19:

19 $$\begin{aligned} \textrm{RMSE} = \sqrt{\frac{1}{N}\sum \limits _{i = 1}^N {{{({x_i}' - {x_i})}^2}} } \end{aligned}$$

where $x'_i$ denotes the value of the chaotic sequence generated with the perturbed initial value, $x_i$ denotes the sequence generated from the original initial value, and *N* represents the sequence length.

**Table 2:** RMSE results of the chaotic system under different initial value perturbations.

Perturbed initial value	X	Y	Z	W
Perturbation $u_x$	7.62643193	8.69512866	11.41662872	4.76635470
Perturbation $u_y$	7.28129330	8.39577590	10.96626276	5.75081103
Perturbation $u_z$	7.44981939	8.58844319	10.51608489	4.95491527
Perturbation $u_w$	7.74941212	8.90794940	10.95912280	5.45109644

The experimental results are presented in Table 2. As observed, even with a minute variation of only $10^{-8}$ in the system’s initial values, the resulting chaotic sequences exhibit marked differences, with the RMSE values for all state variables remaining at a high level. These findings demonstrate that the chaotic system possesses significant sensitivity to changes in initial conditions. Consequently, even extremely small perturbations can lead to a drastic divergence in sequence evolution, thereby effectively enhancing the unpredictability of the key sequence and mitigating security risks associated with initial parameter leakage.

**Fig. 7:**
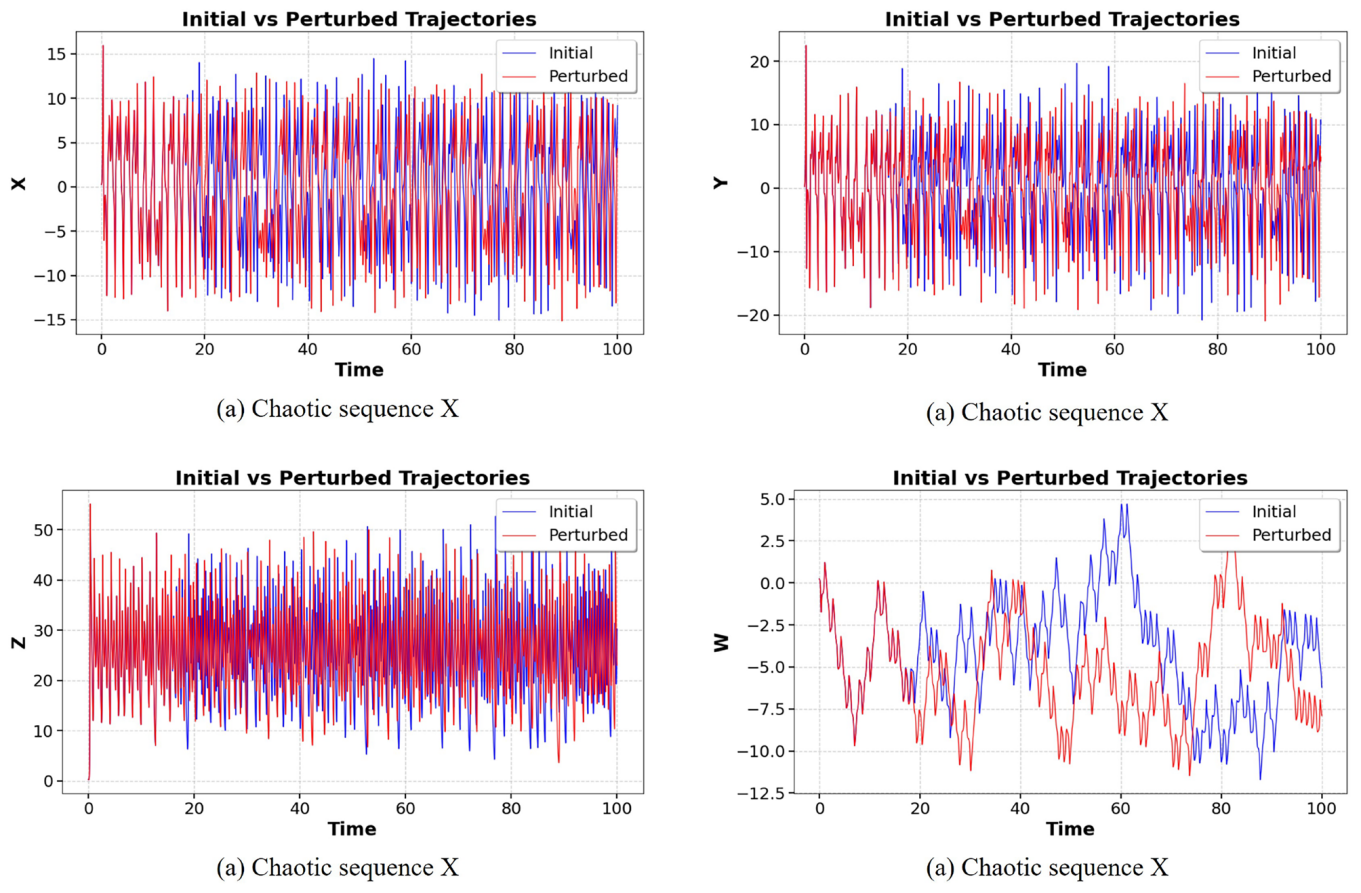
Comparison between original and perturbed trajectories of the chaotic sequence.

To more intuitively observe the impact of initial value perturbations on the system’s evolutionary process, the trajectory variations of the chaotic sequences before and after perturbation (using the perturbation of $u_x$ as an example) are plotted in Fig. 7. It can be seen from Fig. 7 that in the initial stage of iteration, the two trajectories remain largely consistent without obvious differences. However, as the iteration continues, the perturbed trajectory gradually deviates from the original one, eventually exhibiting a completely different evolutionary trend. This phenomenon of rapid trajectory separation caused by minute initial differences is a hallmark manifestation of the sensitive dependence on initial conditions in chaotic systems.

It should be noted that the brief overlap of trajectories in the initial stage represents the transient behavior of the chaotic system, during which the sequences have not yet fully entered a stable chaotic state. Utilizing sequences from this stage for key generation may negatively impact their randomness. Therefore, in the actual encryption process, the initial several iterations of the chaotic sequence are discarded, and only the sequences from the subsequent stable phase are selected to ensure superior statistical randomness.

#### Lyapunov exponent analysis

To further demonstrate the hyperchaotic nature and parameter sensitivity of the proposed four-dimensional chaotic system, a Lyapunov exponent analysis is conducted. Unlike the RMSE-based sensitivity evaluation, which focuses on practical encryption performance, Lyapunov exponents provide a rigorous theoretical measure of chaos by quantifying the average exponential divergence or convergence rate of nearby trajectories in phase space^42^.

For an *n*-dimensional continuous dynamical system, the Lyapunov exponents are defined as

$$\lambda _i = \lim _{t \rightarrow \infty } \frac{1}{t} \ln \frac{\Vert \delta \textbf{x}_i(t)\Vert }{\Vert \delta \textbf{x}_i(0)\Vert }, \quad i = 1, 2, \dots , n,$$

where $\delta \textbf{x}_i(t)$ denotes the evolution of an infinitesimal perturbation along the *i*-th principal direction. A positive Lyapunov exponent indicates sensitive dependence on initial conditions, a hallmark of chaotic behavior. In particular, a hyperchaotic system is characterized by the presence of at least two positive Lyapunov exponents.

Using the system parameters *a=10*, *b=28*, *c=8/3*, *d=1*, and *e=16*, the Lyapunov exponents of the proposed four-dimensional system are numerically estimated after removing transient effects, yielding:

$$\lambda _1 = 0.3943, \quad \lambda _2 = 0.1489, \quad \lambda _3 = -0.0422, \quad \lambda _4 = -14.2025.$$

The presence of two positive Lyapunov exponents ($\lambda _1$ and $\lambda _2$) confirms the hyperchaotic behavior of the system. This property ensures high unpredictability and strong sensitivity to initial conditions, which are essential for cryptographic applications. Moreover, the significant magnitude of the largest negative exponent indicates rapid convergence in one direction while maintaining overall bounded dynamics.

These characteristics indicate that the chaotic system has the potential to generate sequences with high complexity and sensitivity to initial conditions. However, it should be noted that chaotic sequences alone do not necessarily guarantee statistically independent or cryptographically secure randomness without additional processing. In the proposed scheme, the chaotic sequences are not directly used as standalone keystreams, but are incorporated into a dual-layer framework involving coordinate scrambling and SM4-based encryption, which collectively enhances the overall security of the system.

#### Key space

The size and effectiveness of the key space are critical indicators for evaluating the security of an encryption algorithm. The effectiveness of the key space directly determines the system’s resistance to brute-force attacks^43^. In the proposed scheme, the core secret parameter is a 128-bit user key *K*. To further enhance resistance against repeated-key attacks, a 128-bit random nonce *R* is introduced into the initialization process. The user key, random nonce, and map metadata are jointly processed through 32 rounds of SM3 chained iterations to generate the initial states of the 4D hyperchaotic system, scrambling sequences, SM4 keys, and initialization vectors IVs.

Unlike conventional chaotic key space estimation methods that directly treat chaotic initial values as independent entropy sources, the chaotic initial conditions in the proposed scheme are deterministically derived from the user key and therefore should not be regarded as independent secret parameters from a cryptographic perspective. Consequently, the effective brute-force security of the proposed scheme is fundamentally bounded by the entropy of the 128-bit user key, yielding an effective key space of:

$$S = 2^{128}$$

This security level is sufficient to resist exhaustive brute-force attacks using current classical computing technologies. Moreover, even under Grover’s quantum search model, the effective complexity remains approximately $2^{64}$, which still provides practical security for vector geographic data protection^44^. In addition, the introduced random nonce mechanism ensures that different encryption sessions generate completely different chaotic trajectories and ciphertexts even when the same user key is reused, thereby significantly enhancing resistance against repeated-key attacks and ciphertext correlation analysis.

#### Key sensitivity

Key sensitivity analysis is an essential component in evaluating encryption security, measuring how the algorithm responds to slight changes in the key. To verify the key sensitivity of the proposed method, two keys were used: the correct key *K* and an erroneous key *K'* that differs from *K* by only a single bit. After processing through the chaotic system, the SM4 encryption key and IV generated from *K* and *K'* show significant differences, as summarized in Table 3.

**Table 3:** SM4 keys and IVs in key sensitivity analysis.

User Key	SM4 Key	IV
K =	key =	IV =
A1B2C3D4E5F6A7B8C9D0	fb072e3c01df61cdc0c9	de8ef43607af1ae35ca0
E1F2A3B4C5D6	07cc3fa5869c	9f2f14186f9f
K’=	Key’=	IV’=
A1B2C3D4E5F6A7B8C9D0	850beb02303585173b67	9f0658f0e7140a18454d
E1F2A3B4C5D7	053fcc5b6f65	9f542d898135

**Fig. 8:**
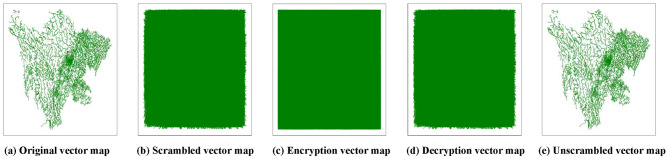
Key sensitivity analysis.

Experimental results indicate that the correct key *K* can successfully decrypt the original vector map (see Fig. 8), whereas *K'*, due to SM4 padding restrictions, fails decryption because of padding errors. These results demonstrate that the proposed method is highly sensitive to even minimal key changes, effectively resisting brute-force and differential attacks, and thereby significantly enhancing the overall security of the system.

### Verification of numerical reversibility in the algorithm

To ensure that the proposed algorithm does not compromise the geometric integrity of vector map data during encryption, numerical reversibility is evaluated from two perspectives: numerical precision and geometric features. Root mean square error (RMSE) and adjacent coordinate correlation (CAC) are employed as quantitative metrics for this validation.

#### Verification of numerical precision

To verify numerical reversibility from a numerical perspective, RMSE is calculated between the original map and the decrypted map. Let the coordinate sequence of the original map be $P = \{ p_1, p_2,..., p_n \}$, and the decrypted sequence be $P' = \{ p_1', p_2',..., p_n' \}$, as expressed in Eq. 20.

20 $$\begin{aligned} RMSE = \sqrt{\frac{1}{\mathrm{{N}}}\sum \limits _{i = 1}^\mathrm{{N}} | |{p_i} - {{p'}_i}|{|^2}} \end{aligned}$$

The closer the RMSE value is to 0, the smaller the numerical error between the two vector maps. Tests were conducted on three types of vector data: London nature, London railway, and London buildings. The results are summarized in Table 4. In all cases, the RMSE between the original and decrypted maps is 0, indicating that the numerical values are perfectly preserved and confirming the algorithm’s numerical reversibility at the numerical level.

**Table 4:** RMSE calculation results.

Data	Number of consistent vertices	Number of inconsistent vertices	RMSE
London nature	189,656	0	0
London railway	111,773	0	0
London buildings	7,512,252	0	0

#### Verification of topological and geometric reversibility

In vector maps, the sequences of x and y coordinates typically exhibit strong spatial topological relationships, manifested as high correlation between adjacent coordinates. An effective encryption algorithm for vector maps should thoroughly disrupt these correlations to enhance security^15^. Researchers commonly evaluate the security of encryption schemes and the reversibility of spatial topology by analyzing the correlation between adjacent coordinates in the original, encrypted, and decrypted maps^37^. To assess both the security and reversibility of the proposed algorithm, we compute the Adjacent Coordinate Correlation (CAC) for the original, encrypted, and decrypted vector maps. The detailed calculation is presented in the following formula:

21 $$\begin{aligned} E(x) = \frac{1}{N} \sum _{i=1}^{N} x_i \end{aligned}$$

22 $$\begin{aligned} D(x) = \frac{1}{N} \sum _{i=1}^{N} (x_i - E(x))^2 \end{aligned}$$

23 $$\begin{aligned} \text {cov}(x, y) = \frac{1}{N} \sum _{i=1}^{N} \big ((x_i - E(x))(y_i - E(y))\big ) \end{aligned}$$

24 $$\begin{aligned} r_{xy} = \frac{\text {cov}(x,y)}{\sqrt{D(x)} \sqrt{D(y)}} \end{aligned}$$

Table 5 presents the adjacent coordinate correlations for line features (London railway) and polygon features (London buildings) across the original map, the encrypted map, and the decrypted map. The analysis shows that in the original maps, the correlation coefficients for both *x* and *y* coordinates are close to 1, indicating strong linear spatial relationships. After encryption, the correlation coefficients drop sharply to near 0, effectively disrupting the original spatial correlation. Upon decryption, the correlations are fully restored to their original values. These results demonstrate that the proposed method effectively reduces inter-coordinate correlations to near zero during encryption (enhancing resistance to statistical and structural attacks) while exactly restoring them upon decryption, thereby achieving full numerical and topological reversibility essential for practical GIS applications.

**Table 5:** Comparison of adjacent coordinate correlations.

Data layer	Adjacent coordinate correlation					
	Original map		Encrypted map		Decrypted map	
	X coordinate	Y coordinate	X coordinate	Y coordinate	X coordinate	Y coordinate
London railway	0.980665	0.977306	0.000859	0.005275	0.980665	0.977306
London buildings	0.994148	0.994999	0.000236	−0.000296	0.994148	0.994999

### Histogram analysis

To evaluate the resistance of the proposed dual-layer encryption scheme against statistical attacks, histogram analysis was conducted using the railway dataset. A secure encryption algorithm should ensure that the encrypted data is uniformly distributed across the entire value range, thereby leaking no information about the original distribution of the coordinates.

**Fig. 9:**
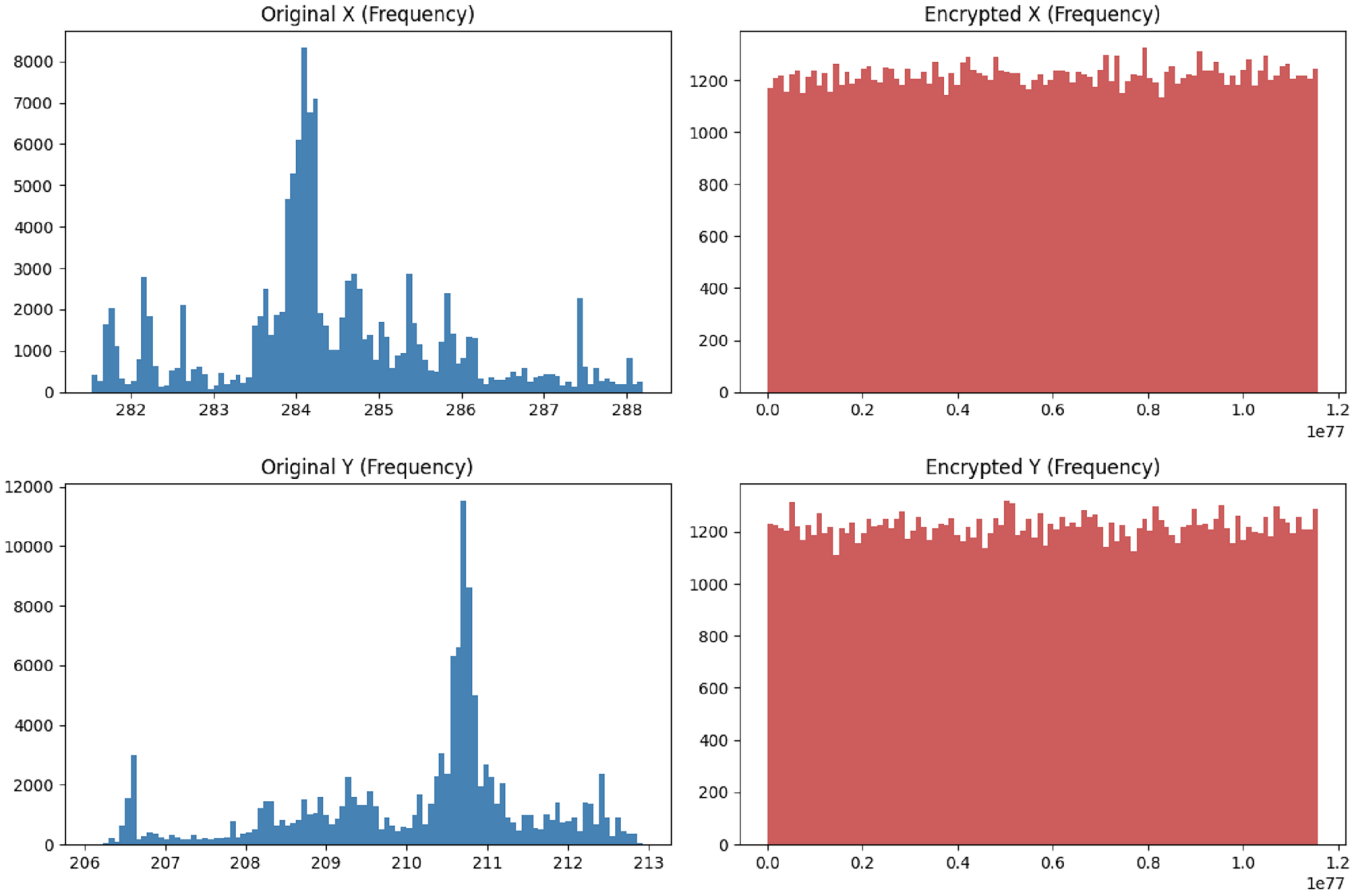
Histogram analysis of *X* and *Y* coordinates for the railway dataset before and after encryption.

Figure 9 illustrates the histograms of the *X* and *Y* coordinates before and after encryption. The original *X* and *Y* coordinates exhibit highly non-uniform distributions, with distinct peaks and clusters that reflect the underlying geographic features of the railway map. In contrast, the histograms of the encrypted coordinates are significantly flattened and exhibit a near-perfect uniform distribution.

This transition from a concentrated distribution to a uniform one demonstrates that the combination of 4D hyperchaotic scrambling and SM4 block encryption effectively obscures the statistical characteristics of the vector data. Even if an attacker captures the encrypted sequence, the uniform railway of the ciphertext prevents any meaningful statistical analysis or pattern recognition, thus confirming the robustness of the scheme against statistical cryptanalysis.

### Cropping attack

To evaluate the robustness of the proposed scheme against data loss during transmission, a cropping attack was performed on the encrypted railway dataset. Continuous segments of the ciphertext, ranging from 10% to 90%, were intentionally removed before decryption.

**Fig. 10:**
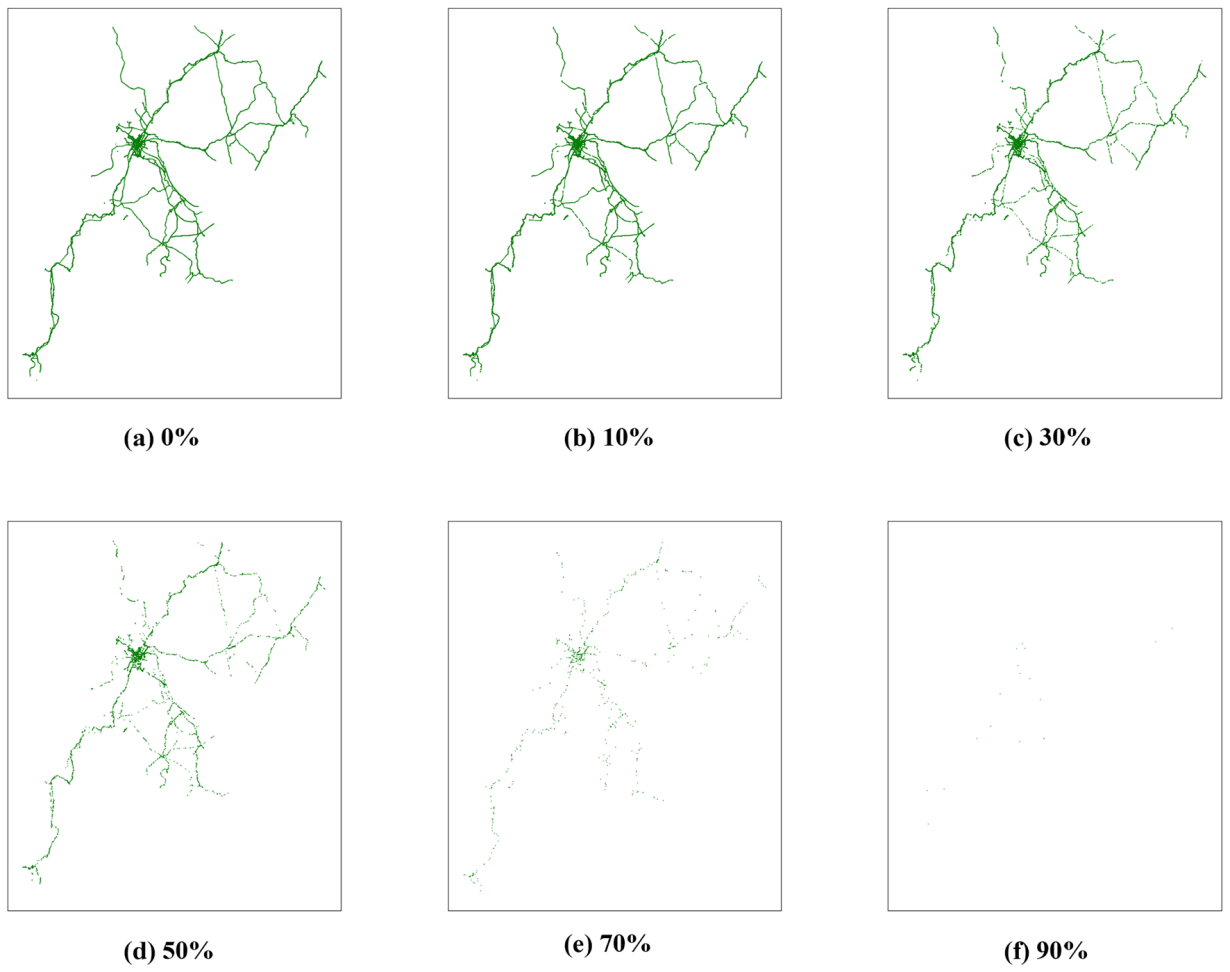
Decryption results of the railway dataset subjected to different proportions of cropping attacks.

Figure 10 illustrates the decryption results under various cropping rates. Thanks to the block-independent nature of the SM4 algorithm and the point-wise scrambling strategy, the proposed scheme effectively prevents error propagation. Even when a significant portion of the ciphertext is lost (e.g., 50% or more), the remaining vertices are still correctly decrypted and re-positioned to their original geographic locations without any precision loss. This experiment demonstrates that the encryption system maintains high data availability and structural integrity for the surviving portions of the vector map even under severe data corruption.

### Information entropy

Information entropy can be used to quantify the randomness of encrypted data. A higher entropy value indicates greater disorder in the data, reducing the likelihood that an attacker can infer the original information. The information entropy *H* is defined as follows:

25 $$\begin{aligned} H = \mathrm{{abs}}\left( {\sum \limits _{i = 1}^{|N|} {\left[ {|K| \cdot {{\log }_2}\left( {\frac{1}{{|K|}}} \right) + |{P_i}| \cdot {{\log }_2}\left( {\frac{1}{{|{P_i}|}}} \right) } \right] } } \right) \end{aligned}$$

where $\text {abs}()$ denotes the absolute value function, *N* represents the data layer composed of points, polylines, and polygons, *K* is the encryption system key, $P_i$ denotes the spatial objects in the vector map layer, and |*K*| and $|P_i|$ represent the cardinalities of *K* and $P_i$, respectively.

In this study, three vector map datasets–Sichuan nature, Sichuan railway, and Sichuan buildings–were selected for experiments. The information entropy of the encrypted data was computed using the proposed algorithm and compared with three existing methods: Yan et al.^16^, Wang et al.^12^, and Tan et al.^14^, denoted as Methods A, B, and C, respectively (see Table 6).

To enable fair comparisons across datasets of different sizes, both absolute entropy and normalized entropy per vertex (absolute entropy divided by vertex count) are reported. The results demonstrate that the proposed method consistently outperforms Methods A, B, and C, achieving 3–6 times higher normalized entropy (e.g., approximately 3 times higher than Methods B and C on most datasets, and up to 6 times higher than Method A on larger datasets). This substantial improvement arises from the dual-layer design: chaotic scrambling disrupts topological relationships, while SM4 encryption introduces high-randomness ciphertext at the coordinate value level–capabilities absent in scrambling-only Method B, DNA-coding-based Method A, or low-dimensional (2D Henon–Sine map) chaos combined with RSA in Method C. Overall, these quantitative results underscore the effectiveness of the proposed scheme in delivering superior randomness and security for vector map data protection.

**Table 6:** Information entropy results (absolute/normalized per vertex).

Data layer	Vertex count	Feature count	Proposed method	Method A	Method B	Method C
			Absolute/normalized	Absolute/normalized	Absolute/normalized	Absolute/normalized
Sichuan nature	6713	6713	517419/77.08	89948/13.40	—	179897/26.80
Sichuan railway	122026	17833	1516429/12.43	256450/2.10	512900/4.20	512900/4.20
Sichuan buildings	937803	114588	11560037/12.33	1930384/2.06	3860769/4.12	3860769/4.12

Method A: Yan et al.^16^; Method B: Wang et al.^12^; Method C: Tan et al.^14^. “—” indicates inapplicability. Normalized entropy = absolute entropy/vertex count.

### Time efficiency

The encryption efficiency of the proposed method was evaluated using the same three vector map datasets (Sichuan nature, Sichuan railway, and Sichuan buildings) and compared with Method A^16^ and Method C^14^.The results are presented in Table 7.

As shown in the table, the proposed method exhibits slightly higher encryption/decryption times than Method A but is substantially faster than Method C, with reductions of approximately 27–47% across the datasets (averaging around 36%). Method A achieves the lowest times through lightweight coordinate scrambling and DNA base mapping, but this lightweight approach provides only coding-level transformation without robust value encryption, resulting in weaker security. Method C, which combines a two-dimensional chaotic map with RSA asymmetric encryption, incurs significantly higher overhead due to large-number modular exponentiation operations in RSA. Method B, relying solely on scrambling, naturally offers the highest speed among the compared approaches; however, it fails to protect coordinate values themselves, leaving the data vulnerable to attacks that exploit unchanged numerical information.

Overall, the proposed dual-layer scheme achieves an effective balance between security and efficiency. The modest increase in computation time relative to lightweight methods is well justified by the substantially enhanced randomness (as evidenced by 3–6 times higher normalized information entropy) and stronger cryptographic protection, making it particularly suitable for applications requiring high security in vector map data.

**Table 7:** Encryption and decryption time efficiency (ms).

Data layer	Size (KB)	Vertex count	Proposed method		Method A		Method C	
			Encryption	Decryption	Encryption	Decryption	Encryption	Decryption
Sichuan nature	184	6713	263	250	97	94	501	511
Sichuan railways	2882	122026	4667	4430	2171	2100	6396	6526
Sichuan buildings	20925	937803	32094	30491	14061	13600	47370	49237

Method A: Yan et al.^16^; Method B: Wang et al.^12^; Method C: Tan et al.^14^. Times are in milliseconds.

### Comparative analysis with AES

To enhance the international relevance of the proposed vector map encryption scheme, we compare the overall framework with AES-128, a widely adopted global cryptographic standard. Table 8 presents the performance comparison between the proposed method and AES-128 in terms of encryption time, root mean square error (RMSE), and information entropy under identical experimental conditions. AES-128 is selected as a representative global cryptographic standard due to its widespread adoption in secure data transmission and storage systems.

As shown in the table, AES-128 demonstrates higher numerical encryption efficiency, resulting in shorter encryption time across point, polyline, and polygon datasets. Both AES-128 and the proposed method preserve numerical precision after decryption, yielding RMSE values of zero for all tested data layers, which confirms the numerical reversibility of both approaches.

However, a clear distinction can be observed in terms of information entropy. Although AES-128 provides effective numerical encryption, its operation is limited to coordinate values and does not alter the spatial topology of vector maps. In contrast, the proposed dual-layer scheme integrates coordinate scrambling with SM4-based numerical encryption, producing ciphertext with significantly higher information entropy. This enhanced randomness reflects the combined effect of structural scrambling and data-dependent key and IV generation, which offers improved resistance against spatial inference and topology-based analysis attacks without introducing prohibitive computational overhead.

**Table 8:** Comparative analysis with AES.

Data layer	Encryption time (ms)		RMSE		Information entropy	
	Proposed method	AES	Proposed method	AES	Proposed method	AES
Sichuan nature	263	158	0	0	517419	86236
Sichuan railway	4667	2893	0	0	1516429	252738
Sichuan buildings	32094	20925	0	0	11560037	1926673

### Computational complexity and resource consumption analysis

To assess the scalability of the proposed scheme in practical GIS environments, we analyze its computational complexity and memory consumption based on the detailed encryption workflow.

Let *|V|* denote the total number of vertices in the vector map. The generation of chaotic sequences is performed using a four-dimensional hyperchaotic system solved by the second-order Runge–Kutta (RK2) method. Although an initial transient phase is introduced to eliminate chaotic degradation, only the final *|V|* values are retained. Since each RK2 iteration involves constant-time numerical operations, the overall time complexity of chaotic sequence generation is *O(|V|)*, and the memory cost of storing four chaotic sequences is linear with respect to *|V|*.

The dynamic chaotic sequence selection relies on simple statistical measures (maximum, minimum, and mean values of coordinate sequences), which are computed in a single pass over the data. The subsequent scrambling process performs index computation and coordinate swapping for each vertex without nested loops or recursive operations, resulting in a linear time complexity.

The SM4 key and initialization vector (IV) are generated by uniformly sampling chaotic sequences. Although each key byte aggregates multiple chaotic elements, the total number of operations across all key bytes is proportional to *|V|*. The SM4 encryption itself applies a fixed number of rounds to each coordinate value, yielding linear computational complexity.

Therefore, the overall time complexity of the proposed dual-layer encryption scheme is *O(|V|)*, and the space complexity is also *O(|V|)*. This linear scalability indicates that the proposed method is well suited for large-scale vector map encryption tasks. Moreover, since different data layers and coordinate sequences can be processed independently, the scheme can be further optimized through parallel or distributed implementations in real GIS systems.

## Discussion

### Discussion on randomness evaluation of chaotic sequences

It should be noted that the chaotic sequences generated in this study are not used as standalone cryptographic keystreams, but rather as auxiliary components for coordinate scrambling and dynamic parameter generation. Therefore, unlike conventional stream cipher designs, the security of the proposed method does not solely rely on the statistical randomness of these sequences. For this reason, standard randomness test suites such as the NIST SP 800-22 are not directly applicable. Instead, the effectiveness of the chaotic sequences is evaluated through sensitivity analysis (RMSE), Lyapunov exponent analysis, and their impact on ciphertext properties such as information entropy and coordinate correlation.

### Cipher integration considerations

In this dual-layer encryption scheme, SM4 was selected over international alternatives such as AES-128 due to its structural compatibility with chaotic key/IV generation, adequate performance, and sufficient security level. Structurally, SM4 and AES-128 both use 128-bit blocks, but SM4’s compact round function (32 nonlinear iterations with simple linear mixing) facilitates straightforward integration of chaotic sequences into key scheduling and IV derivation.Performance-wise, SM4 achieves software efficiency comparable to AES-128 in environments without AES hardware acceleration, which is suitable for large-scale vector coordinate encryption in GIS applications.Security-wise, SM4 provides a 128-bit key space equivalent to AES-128, and no practical attacks against its full 32-round design have been reported to date. Thus, SM4 offers an optimal balance of integration ease, performance, and security for the proposed hybrid approach.

### Resistance to chosen-plaintext attacks

In a chosen-plaintext attack (CPA) scenario, an adversary attempts to infer encryption patterns by submitting carefully constructed plaintexts and analyzing the corresponding ciphertexts. The proposed scheme mitigates such attacks through multiple mechanisms. First, the initial conditions of the hyperchaotic system and the SM4 encryption key and IV are dynamically derived from both the user key and plaintext-dependent features, including file size and coordinate statistics. As a result, different plaintexts lead to distinct chaotic sequences and encryption parameters even under the same user key. Second, the scrambling process is driven by dynamically selected chaotic sequences, making the permutation path highly sensitive to plaintext variations. Finally, the dual-layer design combining structural scrambling and value-level encryption prevents the attacker from establishing a stable plaintext–ciphertext correspondence. These properties collectively enhance the resistance of the proposed scheme against chosen-plaintext attacks.

### Ethical and data governance considerations

Geographic information often contains sensitive spatial features related to infrastructure, transportation, and urban planning. Therefore, encryption schemes for vector maps should not only ensure technical security but also comply with ethical principles and data governance requirements. The proposed method is designed to support secure storage and controlled sharing of geospatial data in accordance with applicable national and international data protection regulations, without altering data semantics or availability for authorized users.

### Limitations and potential extensions

The proposed scheme is primarily designed for two-dimensional vector map data and does not explicitly address three-dimensional GIS datasets or raster data. For 3D vector maps, the framework can be naturally extended by treating the elevation coordinate as an additional sequence and applying the same chaotic scrambling and SM4-based encryption strategy, without modifying the core architecture or affecting topological consistency.

From a practical deployment perspective, the proposed scheme can be integrated into real-time GIS platforms or spatial database systems as a preprocessing or middleware-level security module, enabling secure storage and transmission of sensitive vector map data. Since the scrambling and encryption operations on geometric coordinates are mutually independent, the scheme is inherently suitable for parallel execution. Future work will explore GPU- or FPGA-based acceleration to further improve computational efficiency when processing large-scale or real-time geospatial datasets.

In contrast, raster data differ fundamentally from vector representations, and direct application of the proposed framework is not straightforward. Nevertheless, the chaotic sequence generation and symmetric encryption components could be adapted to pixel- or block-level raster encryption. Extending the proposed framework to support 3D GIS data and more complex application scenarios will be investigated in future research.

## Conclusion

This paper presents a secure vector map data protection scheme that integrates a four-dimensional hyperchaotic system with the Chinese national block cipher SM4, aiming to enhance the confidentiality of vector map data during storage and transmission. The proposed method adopts a dual-layer security architecture that combines structural scrambling with numerical encryption to jointly protect both spatial topology and coordinate values.

Specifically, high-complexity chaotic sequences generated by the four-dimensional hyperchaotic system are first employed to globally scramble the coordinate sequences, effectively disrupting spatial correlations and destroying original topological patterns. Subsequently, the same chaotic sequences are dynamically used to derive the SM4 encryption key and initialization vector, enabling strong value-level encryption of coordinate data and ensuring content confidentiality.

Comprehensive experimental evaluations demonstrate that the proposed scheme exhibits strong resistance against statistical and differential attacks while preserving the topological integrity of vector maps after decryption. The results further indicate that the method supports fine-grained protection of various spatial objects, including points, lines, and polygons, and maintains stable performance across different vector map datasets, making it a promising solution for secure vector map protection in practical GIS environments.

## Data Availability

The datasets generated or analysed during the current study are available in this repository https://github.com/WML991/vector_map_enc.git.
